# Proceedings of the 2019 MidSouth Computational Biology and Bioinformatics Society (MCBIOS) Conference

**DOI:** 10.1186/s12859-020-03580-9

**Published:** 2020-07-06

**Authors:** Jonathan D. Wren, Yongsheng Bai, Zhaohui S. Qin, Da Yan, Ramin Homayouni

**Affiliations:** 1 Genes and Human Disease Research Program, Oklahoma Medical Research Foundation, 825 N.E. 13th Street, Oklahoma City, OK 73104-5005 USA; 2 Biochemistry and Molecular Biology Department, University of Oklahoma Health Sciences Center, Oklahoma City, OK USA; 3 Stephenson Cancer Center, University of Oklahoma Health Sciences Center, Oklahoma City, USA; 4 Department of Geriatric Medicine, University of Oklahoma Health Sciences Center, Oklahoma City, USA; 5 Department of Internal Medicine, University of Michigan Medical School, Ann Arbor, MI 48109 USA; 6 Department of Biostatistics and Bioinformatics, Rollins School of Public Health, Emory University, Atlanta, GA 30322 USA; 7 Department of Computer Science, University of Alabama at Birmingham, Birmingham, AL 35294-1241 USA; 8 Foundational Medical Studies, Oakland University William Beaumont School of Medicine, Rochester, MI 48309-4482 USA

**Keywords:** Bioinformatics, Conferences, MCBIOS, ISCB

## Introduction

The 16th Annual MidSouth Computational Biology and Bioinformatics Society (MCBIOS XVI) conference was held in Hilton Birmingham at University of Alabama Birmingham (UAB) conference center on March 28–30, 2019. The theme of the conference was Informatics for Precision Medicine. The co-chairs and conference hosts were Drs. Jake Y. Chen and Matthew Might from the University of Alabama Birmingham.

The program co-chairs were Dr. Weida Tong from the National Center for Toxicological Research at the Food and Drug Administration (NCTR/FDA) and Dr. Purushotham Bangalore from the University of Alabama Birmingham. The program (detailed below) included two tutorial workshops, 13 breakout oral presentations in seven different scientific tracks, two poster sessions, four keynote speakers, one roundtable discussion, one expert panel session, and two career development sessions. The workshop coordinators were Dr. Andy Crouse from UAB and Dr. Mary Yang from University of Arkansas Little Rock. The student outreach (career development) coordinators were Dr. Inimary Toby from University of Dallas, Dr. Brittany N. Lasseigne from HudsonAlpha, and student representative Ujwani Nukala from University of Arkansas.

A total of 168 people registered for the conference, including 82 professionals, 19 postdoctoral fellows, and 67 students. Among the 157 submitted abstracts, 75 were selected for oral presentations and 41 were selected for poster presentations. The poster session coordinator was Dr. Da Yan from UAB and the chair of the awards committee was Dr. Bindu Nanduri from Mississippi State University.

Executive officers and the new members of the Board of Directors were elected during a business luncheon. The MCBIOS new president this year was Dr. Weida Tong from NCTR/FDA and the President-Elect was chosen as Dr. Jake Y. Chen from UAB. In addition, two new Board members were elected: Drs. Steven Foley from NCTR/FDA and Zhaohui “Steve” Qin from Emory University.

Dr. Cesar Compadre, from the University of Arkansas for Medical Sciences served as the Treasurer and finance coordinator for the conference. The following sponsors provided financial support for MCBIOS XVI. Platinum level sponsors included MCBIOS and the UAB Informatics Institute. Gold level sponsors included the O’Neal Comprehensive Cancer Center at UAB, UAB Center for Clinical and Translational Science, UAB Hugh Kaul Precision Medicine Institute, UAB School of Medicine, and BBVA Compass. Silver level sponsors included Arkansas IDeA Networks of Biomedical Research Excellence and the U.S. Food & Drug Administration.

### Keynote speakers

**Session 1:** “Brain Genomics”, Mark Gerstein, Ph.D., Albert L Williams Professor of Biomedical Informatics, Professor of Molecular Biophysics & Biochemistry and of Computer Science, Co-Director of the Yale Program in Computational Biology & Bioinformatics, Yale University, New Haven, CT.

**Session 2:** “Genomic medicine for understudied populations: lessons from Colombia”, King Jordan, Ph.D., Director of Bioinformatics Graduate Program, Associate Professor of Biology, School of Biology, Georgia Institute of Technology, Atlanta, GA.

**Session 3:** “Semi-Supervised Learning to the Rescue: Towards Deep Understanding of Coding Variant Effects,” Predrag Radivojac, Ph.D., Professor of Computer Sciences, Khoury College of Computer Sciences, Northeastern University, Evanston, IL.

**Session 4:** “Clinical Applications of Genomic Medicine: The State of the Art,” Josh F. Peterson, M.D., MPH, FACMI, Program Director, Masters of Applied Clinical Informatics, Associate Professor of Biomedical Informatics, Associate Professor of Medicine, Vanderbilt University Medical Center, Nashville, TN.

### Workshops

**Tutorial 1:** “Single-Cell genomic data”, Instructor: Min Gao, Ph.D., Informatics Institute, UAB; Co-Instructors: Shanrun Liu, Ph.D., CFCC single cell core, UAB, Jake Y. Chen, Ph.D., Informatics Institute, UAB, Christopher Fucile, MS, Informatics Institute, UAB.

**Tutorial 2:** “Metabolomics Data Analysis”, Instructor: Stephen Barnes, Ph.D., Professor Pharmacology & Toxicology at the University of Alabama at Birmingham (UAB).

### Breakout sessions – oral presentations

**Session 1:** “Integration of Omics Data with Clinical Information,” Session Chair: Chindo Hicks, Ph.D., Louisiana State University School of Medicine, New Orleans, LA; Featured Presentation: “Precision Medicine,” Matthew Might, Ph.D., Hugh Kaul Precision Medicine Institute, UAB School of Medicine, Birmingham, AL.

**Session 2:** “Omics Data in Real-World Settings,” Session Chair: Dong Wang, Ph.D., Senior Statistician, NCTR/FDA, Jefferson, AR; Featured Presentation: “Hierarchical Models for Analyzing Large-Scale Clinical and Molecular Data,” Nengjun Yi, Ph.D., UAB, Birmingham, AL.

**Session 3:** “In Silico Drug Discovery in the Era of Precision Medicine,” Session Chair: Zhichao Liu, Ph.D., NCTR/FDA, Jefferson, AR; Co-moderator: Ting Li, University of Arkansas for Medical Sciences; Featured Presentation: “FDALabel Database on Amazon Cloud with Rich Drug Labeling Information to Advance the Application of Precision Medicine,” Hong Fang, Ph.D., NCTR/FDA, Jefferson, AR.

**Session 4:** “Computer-Aided Drug Discovery and Development,” Session Chair: Daisuke Kihara, Purdue University, West Lafayette, IN; Featured Presentation: “Classification of Ligand-Binding Pockets in Proteins with Deep Learning ,” Michal Brylinski, Ph.D., Associate Professor of Biological Sciences, Louisiana State University, New Orleans, LA.

**Session 5:** “Protein Structural Bioinformatics,” Session Chair: Debswapna Bhattacharya, Ph.D., Department of Computer Science and Software Engineering, Auburn University, Auburn, AL; Featured Presentation: “Computational Protein Structure Modeling for Medium to Low Resolution Cryo-Electron Microscopy Density Maps,” Daisuke Kihara, Ph.D., Department of Biological Sciences and Computer Science, Purdue University, West Lafayette, IN.

**Session 6:** “New Informatics Methods in Precision Medicine,” Session Chair: Steve Qin, Ph.D., Department of Biostatistics and Bioinformatics, Emory University; Featured Presentation: “UALCAN: An Integrated Data Mining Tool for Molecular Sub-Type Based Expression Analysis,” Sooryanarayana Varambally, Ph.D., Department of Pathology, UAB, Birmingham, AL.

**Session 7:** “Microbial Genome Analyses for Public Health,” Session Chair: Steven Foley, Ph.D., Division of Microbiology, NCTR/FDA, Jefferson, AR; Featured Presentation: “Application of Microbiome Sequencing in Food Processing Environments,” Steven Ricke, Ph.D., Center for Food Safety, University of Arkansas, Fayetteville, AR.

**Session 8:** “Challenges and Solutions for Analysis of Gene Expression Data,” Session Chair: Wei Vivian Zhuang, Ph.D., Division of Bioinformatics and Biostatistics, NCTR/FDA, Jefferson, AR; Featured Presentation: “Impact of Pre-Analytical and Analytical Variables in the Quantification of Transcript Levels,” Luísa Camacho, Ph.D., Division of Biochemical Toxicology, NCTR/FDA, Jefferson, AR.

**Session 9:** “Biomedical Informatics,” Session Chair: Wen Zou, Ph.D., NCTR/FDA, Jefferson, AR. Featured Presentation: “Biomedical Informatics: Using Data to Improve Human Health,” Ahmad Baghal, MD, MS, University of Arkansas for Medical Sciences, Little Rock, AR.

**Session 10:** “Heterogeneous Biomedical Information Visualization,” Session Chair: Huanmei Wu, Indiana University Purdue University Indianapolis, IN. Session Co-Chair: Zongliang Yue, UAB, Birmingham, AL. Featured Speaker: “Heterogeneous Biomedical Information Visualization”, Huanmei Wu, Ph.D., Indiana University/Purdue University, Indianapolis, IN.

**Session 11:** “Machine Learning in Biomedicine,” Session Chair: Thanh Nguyen, Informatics Institute, UAB, Birmingham, AL. Featured Speaker: “Interpreting System-Level Cancer Mechanisms Through Rule Learning,” Suping Deng, Ph.D., Department of Electrical and Computer Engineering, Texas A&M University, College Station, TX.

**Session 12:** “Computational Biology,” Session Chair: Stephen Barnes, Ph.D., UAB, Birmingham, AL. Featured Speaker: “New-Generation Single-Cell Mass Spectrometry Tools Enable Trace-level Analysis of Metabolites in the Live Embryo,” Erika Portero, Ph.D., University of Maryland, College Park, MD.

**Session 13:** “Emerging Computational Approaches for Drug Discovery and Development,” Session Chair: Annie Lumen, Ph.D., NCTR/FDA, Jefferson, AR. Student Co-Moderator: Kristin McEuen Ashby, Graduate Student, University of Arkansas at Little Rock, Little Rock, AR.; Featured Speaker: “Lamisil (Terbinafine) Bioactivation Pathways Revealed Through Modeling and Experimental Approaches,” Grover P. Miller, Ph.D., University of Arkansas for Medical Sciences, Little Rock, AR.

## Awards

### MCBIOS young scientist excellence award

MCBIOS Young Scientist Excellence” awards program recognizes students and postdoctoral fellows that exhibit scientific excellence in the field of Bioinformatics. Student and postdoctoral fellows go through a rigorous award application process and the top five candidates are selected to give an oral presentation in a session dedicated to this award program. This was the 3rd year for the “MCBIOS Young Scientist Excellence Award” program.

To compete, students and postdoctoral fellows submitted an abstract and a description of the innovation and their specific contribution to the research. Submissions were first evaluated and ranked by the MCBIOS board members and external judges who evaluated the application for the quality and impact of the research. The top 5 candidates were selected for the postdoctoral and student categories and were invited for an oral presentation in a special session during the second day of the conference. At that time, independent judges evaluated each talk to assess the quality, professionalism, creativity, dedication and multidisciplinary contribution. Judges at each stage of the award process, ensured that there was no conflict of interest (individual, institution etc). Monetary awards for the first, second and third place in student and post-doctoral categories were $200, $150 and $100 respectively. In addition to the monetary award, all the award winners for Young Scientist Excellence Award Winners were presented with an official MCBIOS certificate.

#### Student winners

*First Place*, Hunter Porter, Oklahoma Medical Research Foundation, Oklahoma City, OK “The Biology Behind the Epigenetic Clock.”

*Second Place*, Sutanu Bhattacharya, Auburn University, Auburn, AL. “Does Inclusion of Residue-Residue Contact Information Boost Protein Threading?”

*Third Place*, AyoOluwa Aderibigbe, University of Mississippi, Oxford, MS. “Use of chemoinformatics and molecular docking in the design of peripherally-restricted CB1 antagonists”.

#### Post-doctoral fellow winners

*First Place*, Wenjing Guo, Ph.D., NCTR/FDA, Jefferson, AR. “Development of Software for Facilitating Quality Control of POPs Detection in Food and Animal Feeds”.

*Second Place*, Tanmay Bera, Ph.D., NCTR/FDA, Jefferson, AR. “Improved imaging may help achieve better species level accuracy in identifying food contaminating beetles”.

*Third Place*, Bohu Pan, Ph.D., NCTR/FDA, Jefferson, AR. “Assessment of technical repeatability for germline variants detected from whole genome sequencing (WGS) data”.

### Poster presentation awards

The poster session was held from 4 pm to 5 pm on the first 2 days (March 28 and 29) of the meeting. Student and post-doctoral presenters presented their work at the poster sessions and it was judged for presentation quality by a panel of MCBIOS professional members that attended the conference. Monetary awards of $200 was given for the First place, $150 for Second place and $100 for the Third place.

#### Student winners

*First Place*, Anderson Butler, The University of Alabama at Birmingham, AL. “Context fear memory formation is regulated by hippocampal lncRNA-mediated histone methylation changes.”

*Second Place*, Chase Brown, University of Oklahoma Health and Sciences Center, Oklahoma City, OK. “Bioinformatic detection of synergy for synergistic drug repurposing.”

*Third Place*, Megan Breitbach, Hudson Alpha Institute for Biotechnology & The University of Alabama in Huntsville, AL. “Epigenetic Defects in the B-cell lineage of SLE Patients Display Population-Specific Patterns.”

#### Postdoctoral fellow winners

*First Place*, Vikram Pillai, Ph.D., The University of Alabama at Birmingham, AL. “Hydrogen exchange mass spectrometry reveals allosteric interactions between the distal and the proximal domains of human ADAMTS13.”

*Second Place*, Karen Galarneau, DVM, M.S., Ph.D., Mississippi State University, Starkville, MS. “Transcriptome analysis or understanding host responses to Foot and Mouth Disease Virus.”

*Third Place*, Dongying Li, Ph.D., ORISE Fellow, NCTR/FDA, Jefferson, AR. “Characterization of Non-coding RNA and mRNA Interaction for Gene Regulation in Drug Metabolism and Hepatotoxicity.”

## Selecting papers for the MCBIOS proceedings

The MCBIOS XVI Proceedings contains work presented at MCBIOS 2019, either as an abstract or oral presentation. A total of 19 papers were submitted and 6 were accepted (32% acceptance rate). All papers were anonymously peer-reviewed by at least two reviewers. A summary of the papers is as follows:

Zheng Wang et al., “MASS: Protein Single-model global quality assessment using random forest and newly-designed statistical potentials”. This paper presents a single-model method named MASS for predicting global quality of individual protein models. The authors designed and re-implemented ten protein potentials and proved that these protein potentials are significantly different from each other. Using the values from ten potentials along with six other types of features, a random forest is trained to predict the global quality scores of individual models. MASS was evaluated along with other quality assessment methods in CASP11, CASP12, and CASP13 and the finding is that MASS outperforms most of the methods in CASP11 and is comparable with the leading methods in CASP12 and CASP13.

Hafez Eslami Manoochehri et al., “Drug-Target Interaction Prediction using semi-bipartite graph model and deep learning”. This paper proposes a new framework for drug-target interaction prediction that learns latent features from drug-target interaction network. The problem is modeled as a semi-bipartite graph in which drug-drug and protein-protein similarities are also integrated. For each drug-target pair, an enclosing subgraph is extracted to capture the surrounding environment. Then, a graph labeling method is used for vertex ordering on each enclosing subgraph for adjacency-matrix based encoding. The embedding vectors are then used to train a deep neural network to predict drug-target interactions. The experiments showed that the proposed model can determine interaction likelihood for each drug-target pair and outperform other heuristics.

Mahmut Karakaya et al., Comparison of Smartphone-based Retinal Imaging Systems for Diabetic Retinopathy Detection using Deep Learning. The authors investigate the smartphone-based portable retinal imaging systems available on the market and compare their image quality and the automatic DR detection and the automatic DR detection accuracy using a deep learning framework. The authors observed that the network DR detection performance decreases as the field of view of the smartphone-based retinal systems get smaller where iNview is the largest and iExaminer is the smallest. The smartphone-based retina imaging systems can be used as an alternative to the direct ophthalmoscope.

King Jordan et al., Ancestry effects on type 2 diabetes genetic risk inference in Hispanic/Latino populations. The authors investigated how ancestry affects the inference of T2D genetic risk using PRS in diverse HL populations from Colombia and the United States (US). In Colombia, the authors compared T2D genetic risk for the Mestizo population of Antioquia to the Afro-Colombian population of Chocó, and in the US, they compared European-American versus Mexican-American populations. The experiments showed that T2D genetic risk in these HL populations is positively correlated with African and Native American ancestry and negatively correlated with European ancestry. The inferred relative risk of T2D is robust to differences in the ancestry of the cohorts used for variant discovery. Adam Thrash et al., Toward a More Holistic Method of Genome Assembly Assessment.

Assembly of full genome sequence is a key application of the high throughput sequencing technology. Traditionally, genome assemblies are assessed using statistics relating to contiguity of the assembly. In this paper, thrash et al. presents a review of problems that arise from relying solely on contiguity as a measure of genome assembly quality as well as current alternative methods. Alternative methods are compared on the basis of how informative they are about the biological quality of the assembly and how easy they are to use. A comprehensive method for using multiple metrics of measuring assembly quality is presented. Weaknesses and strengths of varying methods are presented and explained, with recommendations based on speed of analysis and user friendliness. The authors also offer a comprehensive method that incorporates multiple facets of quality assessment.

Yongsheng Bai et al., “MMiRNA-Viewer^2^, a Bioinformatics Tool for Visualizing Functional Annotation for MiRNA and MRNA Pairs in a Network”. This paper presents an innovative bioinformatics tool, MMiRNA-Viewer^2^, for visualizing functional relationships between miRNA and mRNA pairs in a network utilizing the next generation sequencing data of miRNA and mRNA expression profiles in tumor and normal samples. The tool fills the gap that existing tools can not characterize and visualize functional consequences of cancer risk gene and miRNA pairs while analyzing the tumor and normal samples simultaneously. The tool takes mRNA and miRNA interaction pairs to display the mRNA and miRNA gene annotation information, signaling cascade pathways and direct cancer association between miRNAs and mRNAs. Functional annotation and gene regulatory information can be directly retrieved from the tool web server, which can help users quickly identify significant interaction sub-network and report possible disease or cancer association. The tool is applicable across a range of diseases and cancers and has advantages over existing tools.

## Future meetings

The 17th Annual MCBIOS conference will be hosted by the SAS Institute in Cary, NC on April 26-28, 2021. The conference co-chairs are Drs. Weida Tong and Steven Foley from NCTR/FDA, and Dr. Inimary Toby from University of Dallas, TX.

## Data Availability

Not applicable.

